# Gut–Brain Signaling in Parkinson’s Disease: A Narrative Review

**DOI:** 10.3390/ijms27083531

**Published:** 2026-04-15

**Authors:** Karolina Ratajczyk, Emilia Kaczorowska, Karolina Wyka, Aleksandra Tarasiuk-Zawadzka, Jakub Fichna, Agata Gajos

**Affiliations:** 1 Department of Neurology, Karol Jonscher’s Municipal Medical Center, Milionowa 14, 93-113 Lodz, Poland; karolina.j.ratajczyk@gmail.com; 2 Department of Biochemistry, Faculty of Medicine, Medical University of Lodz, Mazowiecka 5, 92-215 Lodz, Poland; emilia.kaczorowska@student.umed.lodz.pl (E.K.); karolina.wyka@umed.lodz.pl (K.W.); aleksandra.tarasiuk-zawadzka@umed.lodz.pl (A.T.-Z.); jakub.fichna@umed.lodz.pl (J.F.); 3 Department of Extrapyramidal Diseases, Medical University of Lodz, Pomorska 251, 92-213 Lodz, Poland; 4 Department of Neurology, Central University Hospital, Pomorska 251, 92-213 Lodz, Poland

**Keywords:** parkinsons disease, microbiota, gut-brain axis, neurotransmitters

## Abstract

The formulation of the gut–brain–microbiota axis (GBA) theory has led to new research directions that have expanded our understanding of the pathogenesis, phenotypic variability, and clinical course of Parkinson’s disease (PD). Models of PD pathogenesis, based on the Braak hypothesis, suggest a subtype of the disease in which pathological changes begin in the gut many years before the onset of brain pathology and the manifestation of motor symptoms. Gut microbiota may influence nervous system function along the GBA by influencing intestinal permeability, chronic inflammation, and α-synuclein aggregation. Accumulating evidence suggests that the gut microbiota may also regulate the synthesis and metabolism of neurotransmitters, including dopamine (DA), serotonin (5-HT), acetylcholine (ACh) and γ-aminobutyric acid (GABA), both in the gut and brain, and indirectly stimulate central nervous system activity via the vagus nerve, which receives signals from the enteric nervous system. Research on the effects of microbiota on GBA has paved the way for the identification of novel treatment strategies, including probiotics, prebiotics, synbiotics, postbiotics, antibiotics, and fecal microbiota transplantation (FMT), aimed at not only symptomatic but also disease-modifying treatment of PD. In this article, we propose a novel approach to GBA as a link between gut microbiota and gut and brain neurotransmitter metabolism in PD. We review the latest research on the gut epithelial barrier. We analyze and summarize the potential of therapeutic interventions targeting gut microbiota and their impact on neurotransmitter regulation in PD.

## 1. Introduction

Parkinson’s disease (PD) is the second most common neurodegenerative disorder after Alzheimer’s disease [1,2,3]. The classic motor symptoms of PD include bradykinesia, limb rigidity, and resting tremor. In addition to motor symptoms, non-motor manifestations, such as autonomic symptoms, sleep disturbances, pain, and neuropsychiatric symptoms, are also frequently present in PD. Most of these symptoms appear even before the motor symptoms and have a serious impact on the quality of life of patients [4]. Constipation and other GI disorders such as dysphagia, gastroparesis, nausea, and heartburn are among the most common and earliest symptoms of PD [5]. The neuropathological hallmark of PD is the presence of Lewy bodies—intracellular clusters of abnormal, insoluble α -synuclein—in both the central and peripheral nervous systems. α-Synuclein lesions consist of aggregates of α-synuclein fibrils with an abnormal tertiary structure. The α-synuclein protein undergoes abnormal folding and aggregates into eosinophilic inclusion bodies, also known as Lewy bodies. The main component of Lewy bodies is a cytoplasmic protein consisting of 140 amino acids, which is encoded by the α-synuclein (SNCA) gene. α-Synuclein is chemically stable and is not easily hydrolyzed by its own enzymes [6]. The discovery of α-synuclein deposits in the ENS of patients with Parkinson’s disease [7] and the subsequent hypothesis by Braak et al. [8] that synucleinopathy originates in the DMNV and the anterior olfactory nucleus led to the development of an ‘ascending anatomical theory’ of the transmission of PD pathology from the gut to the brain. This theory marked a milestone in PD gut–brain axis research and established the gastrointestinal tract’s role in this disease’s pathogenesis. Pathogenic factors affecting the gut induce α-synuclein misfolding and aggregation, beginning in the enteric nervous system (ENS) and propagating to the brain via cell-to-cell transfer, akin to prion transfer [9]. The gut–brain hypothesis has profoundly influenced the concept of PD pathogenesis [8,10] and contributed to Horsager et al. [11] proposal of two PD subtypes: a top-down brain type, in which α-synuclein pathology initially arises in the brain and then spreads to the peripheral autonomic nervous system, and a bottom-up body type, in which the pathology originates in the enteric or peripheral autonomic nervous system and then spreads to the brain. Although numerous contradictory results supporting and opposing this hypothesis have been reported in subsequent years in both experimental and observational studies, the current decade has seen a wealth of evidence pointing to impaired intestinal permeability (‘leaky gut’) and inflammation in PD [12,13,14]. The most important neuropathological feature of PD historically was the degeneration of the substantia nigra pars compacta (SNc). It is currently widely accepted that the spread of α-synuclein in the brain occurs in stages, with damage to other areas preceding the degeneration of substantia nigra compacta (SNc) neurons [8,15]. According to Braak’s theory [16], α-synuclein originating from changes in the enteric nervous system (ENS) diffuses through the gut–brain axis (GBA) to the dorsal motor nucleus of the vagus nerve. It then spreads through the olfactory bulb to the central nervous system CNS and occupies the locus coeruleus and substantia nigra. Subsequently, it extends to cortical areas, thereby causing PD [8]. It has been reported that the risk of developing PD is lower if a complete vagotomy (stem vagotomy) is performed five years before the onset of the disease. Selective vagotomy, as well as not undergoing surgery, is associated with an almost identical risk of developing PD [17,18]. The gut microbiota regulates GI physiology through interactions in the intestinal environment between the intestinal epithelium, the mucosal immune system, and the ENS. The ENS is an extensive network of neurons and glial cells modulated by signals from the CNS; however, it is also capable of autonomous functioning. Communication between the ENS and the CNS is bidirectional. This communication takes place via GBA, which is an interactive network connecting the gut to the brain, regulated by the microbiome as well as by immune, neuroendocrine, and neuronal mechanisms [19]. The gut microbiome is a key component of the GBA that can influence CNS homeostasis by modulating the immune system. Dysbiosis, defined as changes in the composition of the gut microbiota that disrupt its balance, is associated with GI dysfunction, CNS inflammation, and neurodegeneration [20]. Bacteria, fungi, archaea, viruses, and parasitic worms in the intestines form a stable microbiological environment of over 100 trillion microorganisms. The gut microbiome is first colonized at birth. The development of microbiome homeostasis in infants is influenced by maternal obesity and diet composition [21]. The number and diversity of the gut microbiome increase during the first five years of life and then stabilize with age [22]. Furthermore, factors such as stress, infection, diet, lifestyle and geography can modify the homeostasis of the gut microbiome [23]. The gut microbiota affects the metabolism and function of various neurotransmitters, including dopamine (DA), serotonin (5-HT), acetylcholine (ACh), γ-aminobutyric acid (GABA), glutamate, and norepinephrine (NE), thereby influencing signaling within GBA. The gut microbiota influences neurotransmitters in the gut and brain by directly participating in their synthesis, affecting the enzymes that regulate the rate of their main metabolic reactions, or interacting with the transporters of certain receptors and neurotransmitters [24,25].

In this paper, we describe GBA. Our focus is on the impact of gut microbiota on PD and associated disturbances in the metabolism of gut neurotransmitters DA, 5-HT, ACh and GABA. We analyze their potential impact on the pathogenesis and progression of PD and also review the latest research findings on the intestinal epithelial barrier, emphasizing its pivotal role in gut–brain signaling and the processes of neuroinflammation and neurodegeneration. Finally, we summarize the potential of gut microbiota-targeted therapeutic interventions in the treatment of neurodegeneration, with a particular focus on PD. This review aims to inspire further research into the GBA, particularly with regard to the significance of gut neurotransmitter dysregulation for the pathophysiology of PD.

To prepare this paper, a literature review was conducted in the PubMed and Scopus databases. Inclusion criteria included original and review articles published in English between 1996 and 2026. The keywords and phrases used in the search were: Parkinson’s disease, gut–brain axis, microbiota, dysbiosis, neurotransmitters, dopamine, sertraline, GABA, acetylcholine, enteric glia, enteric nervous system, probiotics, prebiotics, synbiotics, postbiotics, and fecal microbiota transplantation. Conference proceedings, case reports, and articles without full text were excluded from the analysis.

## 2. The Influence of Intestinal Microbiota on the Development and Permeability of the Intestinal Epithelial Barrier

The development, maturation, and continuous renewal of the ENS are strongly influenced by the gut microbiota, particularly microorganisms capable of producing or metabolizing neuroactive compounds. The digestive tract and brain are formed from parts of the embryo that are closely related [26]. The enteric nervous system (ENS) is frequently designated as the ‘second brain’. The GI tract and the brain are characterized by the presence of specific, specialized vascular barriers, namely the blood–brain barrier (BBB) and the intestinal epithelial barrier. The BBB is primarily composed of capillary endothelial cells (ECs). The EC of the BBB is a component of the ‘neurovascular unit,’ which also consists of neurons, extracellular matrix, pericytes, and astrocytes [27,28,29]. The majority of intestinal neurons and the intestinal epithelial barrier are developed during the early intrauterine life stage, with this process continuing into the early postnatal period. During development, a subset of neural crest cells expressing Sox10 migrates to the foregut, proliferates, and ultimately colonizes the entire digestive tract, differentiating into neurons and glial cells. Microbiological colonization is essential for normal neurogenesis of the nervous system. In murine models, recolonization of the GF intestine induces the expression of nestin, an intermediate filament protein involved in regulating the balance between apoptosis and neuron regeneration, and Ki67, a nuclear marker associated with rRNA transcription and cell proliferation. Reduced expression of either of these proteins reflects impaired health of neural stem cells and reduced ability to regenerate neurons. Importantly, nestin is also expressed in the placenta and serves as a marker of neuronal integrity in the developing central nervous system [30]. Studies involving germ-free (GF) newborn mice also indicate that the gut microbiota plays a direct role in shaping the postnatal development of the enteric nervous system (ENS). In particular, microbial colonization appears to influence GI motility by modulating both the total number of intestinal neurons and the relative abundance of specific neuron subtypes [31]. The sealing of ECs (endothelial cells) is facilitated by tight junction proteins (TJ, occlusive junctions, zonula occludens) within the cells. These proteins contribute to the regulation of the integrity and function of the BBB (blood–brain barrier) [27,28,32]. TJs limit the paracellular diffusion of water-soluble substances from the blood into the brain parenchyma, ensuring the proper functioning of neurons [22]. An intact BBB protects newborns at a critical stage of development from colonization by microbiota, and in the postnatal period also protects against bacterial metabolites and exposure to new molecules during metabolic switching (from carbohydrates to fatty acid catabolism) [28,33].

## 3. Role of Enteric Glia in Enteric Nervous System Homeostasis

Intestinal glia play an important role in GI disorders associated with PD. Enteric glial cells (EGC) are an integral part of the enteric nervous system (ENS). They share many morphological, molecular, and electrophysiological features with brain glial cells, particularly astrocytes [34]. In addition to mechanical, immunological, and homeostatic support, EGCs perform neurotransmitter functions and participate in epithelial barrier integrity, epithelial cell proliferation and differentiation, and intestinal defense [35]. EGC cells originate from bipotent neural crest progenitor cells that disseminate throughout the GI tract, exhibiting phenotypic diversity that is associated with their adaptation to variations in the microenvironment (e.g., diverse microbiota) [36]. The primary function of intestinal glia is to maintain the homeostasis of the enteric nervous system (ENS), especially by influencing intestinal neural reflexes and neuroinflammation. This strong bidirectional communication occurs in neuron–glia connections. In addition to receiving neurotransmitters from the ENS, the intestinal neuroglia produces glia transmitters such as ATP and GABA, as well as paracrine mediators that activate ascending excitatory pathways or slow down descending inhibitory pathways through interaction with receptors [37]. EGC forms a network that communicates with the plexus and all layers of the intestinal wall. Similar to astrocytes, they act as a functional syncytium, promoting the propagation of calcium waves in response to various neurotransmitter stimuli, including ATP, serotonin (5-HT), and acetylcholine (ACh) [38]. It has been well documented that EGCs play a role not only in neuronal neurotransmission, through the degradation or sequestration of neurotransmitters released by synapses, but also in the production of neurotransmitter precursors, e.g., L-arginine or glutamine [39].

## 4. Gut Dysbiosis in Parkinson’s Disease

Inflammation of the intestines and intestinal barrier dysfunction resulting from intestinal dysbiosis are risk factors for PD in genetically susceptible hosts [40]. Distinctive changes in the gut microbiota of PD patients can be used as early biomarkers of the disease [41]. Compared to fecal microbiota transplantation (FMT) from healthy donors, transfer of gut microbiota from patients with PD exacerbates motor deficits and pathological changes in mice overexpressing α-synuclein [5]. Among mouse models of PD with identical genetic backgrounds and overexpression of α-synuclein, germ-free (GF) mice showed significantly better motor performance compared to mice with conventional microbiota. Importantly, both motor disorders and abnormal α-syn aggregation were alleviated after depletion of the gut microbiota. Furthermore, gut microbiota profiles strongly correlate with disease duration, age of onset, and severity of motor and non-motor PD symptoms [42,43]. In comparison with healthy subjects, patients suffering from PD have been shown to exhibit differences in the diversity of beta and alpha microbiota, in addition to alterations in taxonomic composition [44]. At the genus level, the most common changes include increases in *Christensenella*, *Oscillospira*, *Clostridium*, *Enterococcus*, *Streptococcus*, *Lactobacillus, Parabacteroides*, *Alistipes*, *Bifidobacterium*, *Escherichia*, *Desulfovibrio*, *Bilophila*, and *Akkermansia*, along with decreases in *Blautia*, *Roseburia*, *Fusicatenibacter*, *Streptococcus*, *Faecalibacterium*, *Prevotella*, and *Haemophilus* [45]. Numerous studies consistently report a reduction in butyrate-producing genera such as *Faecalibacterium* and *Roseburia*, along with increased abundance of *Akkermansia* and *Bilophila*, as microbiological signatures of PD [46]. *Lactobacillus*, *Bifidobacterium*, and *Akkermansia muciniphila* are beneficial gut bacteria. Elevated levels of these bacteria are associated with the presence of additional modifying factors. Li et al. [47] found that increased numbers of *Bifidobacteriaceae* as well as *Lactobacillaceae* showed significant correlation with clinical indicators of inflammation, such as neutrophil percentage, monocyte percentage, and monocyte count.

Increases in *Bifidobacterium* and *Lactobacillus* are often associated with the use of anti-Parkinson’s drugs, in particular COMT (catechol-O-methyltransferase) inhibitors. In contrast, an increase in *Akkermansia* levels has been observed during the ageing process [45].

*Akkermansia muciniphila* has also been demonstrated to degrade the intestinal mucus layer, thereby increasing intestinal permeability and potentially driving inflammation and oxidative stress [48]. However, reduced levels of Prevotellaceae in PD are associated with improved barrier integrity through mucin production [49].

**Figure 1:**
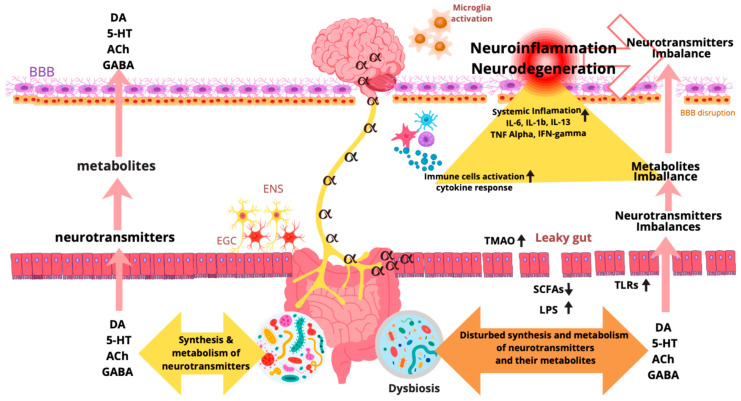
The impact of dysbiosis on the gut–brain axis in Parkinson’s disease. Intestinal dysbiosis may contribute to the onset and progression of PD by affecting the gut–brain–microbiota axis (GMBA), resulting in damage to the intestinal epithelial barrier, increased intestinal permeability (leaky gut), and exposure of the enteric nervous system (ENS) to proinflammatory bacteria, their products, and inflammatory mediators. Blood–brain barrier (BBB) disruption may occur as a result of gut bacterial products, such as lipopolysaccharides (LPS), or peripheral inflammatory responses, such as cytokine production. Models of PD pathogenesis based on the Braak hypothesis suggest a subtype of the disease in which pathological changes begin in the gut. Accumulation of α-synuclein (α) may begin in the ENS before spreading via GMBA to the CNS. Gut microbiota can also regulate the synthesis and metabolism of neurotransmitters: dopamine (DA), serotonin (5-HT), acetylcholine (ACh), γ-aminobutyric acid (GABA), and norepinephrine (NE), both in the gut and indirectly in the brain. Other abbreviations in the figure: enteric glial cells (EGC), short-chain fatty acids (SCFAs), trimethylamine N-oxide (TMAO), Toll-like receptors (TLRs).

At the species level, most studies observed a decrease in *Blautia wexlerae*, *Faecalibacterium prausnitzii*, and *Roseburia* in PD patients, while *Clostridium leptum*, *Bifidobacterium bifidum*, *Bifidobacterium dentium*, *Eisenbergiella tayi*, *Lactobacillus salivarius*, and *A. muciniphila* were increased. Furthermore, a higher prevalence of the species *Ruthenibacterium lactatiformans*, which has received only limited study, was also reported. This species demonstrates a high degree of genetic similarity to *Faecalibacterium prausnitzii*, and it is hypothesized that competition between the two species may underlie the observed associations with PD [50]. Intestinal dysbiosis may contribute to the onset and progression of PD by damaging the intestinal epithelial barrier, increasing intestinal permeability, and exposing the enteric nervous system (ENS) to pro-inflammatory bacteria, their products, and inflammatory mediators (Figure 1). Blood–brain barrier (BBB) disruption may occur as a result of the action of intestinal bacterial products (e.g., lipopolysaccharides, LPS) or peripheral inflammatory reactions (e.g., cytokine production) [43,51]. The pro-inflammatory effect in the pathogenesis of PD is mainly attributed to lipopolysaccharides (LPS)–endotoxins that are part of the outer membrane of Gram-negative bacteria, e.g., *Escherichia coli*, protecting them and stimulating a strong immune response in the host [45,52]. LPS activate TLR4 signaling, leading to increased cytokine production, oxidative stress, and microglial activation. Both endotoxins and intestinal dysfunction raise LPS levels in both the intestines and blood of PD patients, thereby promoting α-synuclein aggregation in intestinal neurons and causing peripheral–and subsequently systemic–inflammation [53,54]. Metabolites derived from the gut microbiota and their receptors, apart from the immune system, maintain metabolic homeostasis, which is essential for balancing the utilization and consumption of nutrients. SCFAs such as butyrate, acetate, and succinate are produced as a result of bacterial fermentation in the gut [55]. In PD patients, a significant decrease in the abundance of SCFA-producing gut bacteria, including *Faecalibacterium*, *Lactobacillaceae*, *Ruminococcaceae*, *Eubacterium*, *Bacteroides*, and *Prevotella*, has been observed, accompanied by reductions in SCFA levels, such as butyrate, acetate, and propionate concentrations [56]. SCFAs contribute to improving the function and stability of the intestinal mucosal barrier by stabilizing hypoxia-inducible factor (HIF) and activating AMP-activated protein kinase (AMPK), providing energy to epithelial cells and regulating the expression of tight junction proteins (TJP) [57,58]. SCFAs may also strengthen the intestinal barrier by stimulating mucus production through activation of Free Fatty Acid Receptor 3 (FFAR3) [59]. SCFAs reduce neuroinflammation in PD by strengthening the intestinal barrier and reducing the penetration of inflammatory factors, bacterial products, and α-synuclein into the systemic circulation [60]. SCFAs may also influence the composition and structure of the intestinal microbiota by modifying intestinal pH, mucosal permeability, mucin synthesis, and intestinal immunity. Sodium butyrate supplementation effectively improved gut dysbiosis in rotenone and MPTP models and helped establish a new gut microbiota balance [61], suggesting a regulatory interplay between microbial metabolites and gut microbiota. Furthermore, SCFAs can maintain BBB integrity by interacting with FFAR3 receptors on brain endothelial cells. Propionate, on the other hand, can reduce cell surface CD14 expression and activate nuclear factor erythroid 2-related factor 2, thereby reducing BBB damage caused by oxidative stress [62].

The gut microbiota synthesizes numerous neurotransmitters present in the human brain. Dysbiosis of the gut microbiota can disrupt the synthesis of neurotransmitters, including dopamine and 5-hydroxytryptamine, acetylcholine, GABA, and NE.

This disruption of CNS homeostasis through GBA signaling pathways may contribute to the pathological progression of neurological dysfunction in PD [63]. Gao et al. [64] revealed that after altering the gut microbiota in experimental animals through antibiotic infusion, there was a significant reduction in serotonin, DA, and aromatic amino acid concentrations in the blood and hypothalamus compared to the control group, which was administered normal saline solution. Similarly, van Kessel et al. [65] investigated the effect of bacterial tyrosine decarboxylase in the proximal small intestine–the main area of L-DOPA absorption–in patients with PD. They observed an increase in bacterial tyrosine decarboxylase activity in the proximal small intestine (the main area of L-DOPA absorption) in PD patients, leading to premature L-DOPA conversion, significantly reducing its plasma concentration and bioavailability. This, in turn, increased the therapeutic dose requirement and reduced the efficacy of the drug. According to these reports, intestinal dysbiosis may directly or indirectly affect the pharmacokinetics, bioavailability, and adverse effects of drugs used to treat PD [66]. It is important to consider that most current evidence in humans comes from observational studies, which demonstrate consistent correlations between dysbiosis and neurodegenerative pathology but are limited in establishing a causal relationship. No single species of bacteria, viruses, fungi, or archaea has been identified as the sole causative factor for neurodegenerative diseases. However, drug-induced changes in the gut microbiome may be a key factor in the pathogenesis of PD. Long-term use of macrolide and tetracycline antibiotics has been observed to significantly alter the composition of the gut microbiota, potentially contributing to the development of PD [67]. On the other hand, the use of therapeutic interventions targeting the microbiome may represent a promising avenue for research and clinical application. Geographic, environmental, and dietary factors may also influence the composition of the gut microbiota [68,69,70].

## 5. Microbiota–Gut–Brain Axis and Neurotransmitters (Interaction Between Microbiota and Neurotransmitters)

The gut microbiota plays a crucial role in shaping the synthesis, metabolism, and function of neurotransmitters, including DA, 5-HT, Ach, and GABA, which in turn influences bidirectional gut–brain signaling (Table 1). The precise mechanisms by which the gut microbiota influences neurotransmitter levels in the gut and central nervous system are not yet fully understood. As indicated by extant literature, these pathways may comprise direct microbial synthesis of neurotransmitters, modulation of enzymatic steps that limit their metabolism, and changes in the expression or activity of neurotransmitter transporters [25,67].

### 5.1. Dopamine and Influence of Gut Microbiota on Dopamine and L-DOPA Metabolism

DA has been identified as playing a pivotal role in the CNS. Within the brain, dopamine comprises approximately 80% of catecholamine content and is synthesized locally by neurons from four main axonal pathways: nigrostriatal, mesolimbic, mesocortical, and tuber-funicular. The nigrostriatal pathway regulates motor control, connecting the compact part of the substantia nigra with the dorsal striatum in the forebrain. The mesolimbic pathway is responsible for reward and pleasure-related behaviors, transmitting dopamine from the ventral tegmental area (VTA) in the midbrain to the ventral striatum [80]. The mesocortical pathway, which influences cognitive functions and emotional behaviors, connects the VTA to the prefrontal cortex. The tuber–infundibular pathway regulates prolactin secretion and connects the arcuate nucleus with the median eminence in the hypothalamus [80].

Outside the CNS, DA is also produced locally in several peripheral organs, including the GI tract, smooth muscle, mesenteric vessels, coronary vessels, renal vessels, and the kidneys, where it acts (autocrine and paracrine) as a local hormone [81]. Physiological studies have shown that almost half of the dopamine in the body is produced in the intestines. Its receptors are widespread in the GI tract, where they influence gastric secretion, motility, and blood flow through the mucosa [82,83]. It has been found that neurons throughout the human digestive tract, both in the submucosal plexuses and in the myenteric plexus, express tyrosine hydroxylase [84]. This suggests that they have the ability to synthesize dopamine. Dopamine probably has independent bioactivity in various organs. In the heart, there is a rapid conversion of dopamine to noradrenaline by sympathetic neurons, whereas in the intestines and mesenteric vessels, this is less likely due to the high ratio of dopamine to noradrenaline. Dopamine is synthesized via the biochemical pathway phenylalanine–tyrosine–L-DOPA–dopamine. Both tyrosine and L-DOPA are capable of crossing the blood–brain barrier; however, L-DOPA is relatively unstable and undergoes rapid decarboxylation to dopamine in the small intestine by aromatic L-amino acid decarboxylase [85]. Dopamine itself is unable to cross the blood–brain barrier; however, the transmission of signals via dopamine between the brain and the gut, including via the gut microbiome, is a powerful regulator of both the central nervous system and GI pathology [85]. The gut microbiota not only influences dopamine regulation but also contributes directly to its production and the integrity of dopaminergic pathways and can modulate both L-DOPA availability and dopamine production in the host [84,86]

PD is mainly associated with the regulation of dopaminergic signal transmission via D1–D3 receptors, which control locomotor activity [87]. All five types of dopamine receptors (D1–D5) are present in the peripheral nervous system. Their expression varies depending on their location within the GI tract. These receptors mediate a wide range of functions, including strengthening mucosal integrity (D1 and D2) and modulating motility (D2) [87].

Gastroparesis, as a potential predictor of PD, unlike peptic ulcer disease (PUD), which is also associated with PD risk, may indicate peripheral dopamine excess (rather than deficiency, as in PUD) [88]. Gastroparesis is more commonly observed after a PD diagnosis and may be, at least in part, iatrogenic. Gastroparesis as a side effect of L-DOPA therapy is well documented. This is largely due to the fact that L-DOPA undergoes peripheral metabolism to dopamine before crossing the blood–brain barrier. However, gastroparesis can also manifest in the prodromal phase of PD (before the start of dopaminergic therapy) and cannot be attributed to exogenous L-DOPA. Instead, it may be associated with excessive peripheral dopamine production [89,90,91]. Dopaminergic immunomodulation plays a role in both innate and acquired immunity. It is believed to be largely mediated by the activity of MAPK and AKT kinases [92,93,94]. Human macrophages exhibit high levels of D1 and D2 receptor expression and lower levels of other dopamine receptors [95]. In contrast, T lymphocytes tend to express all dopamine receptors at lower levels [96]. Dopamine is likely to have a significant impact on neutrophil activity. Observations indicate that, at higher doses in humans, it compromises the capacity of neutrophils to adhere to the endothelium and increases their apoptosis [97]. In a study on rats, the opposite situation was observed. At lower doses, dopamine increased the number of neutrophils [98]. Immune cells themselves appear to produce small amounts of dopamine, as evidenced by experiments inhibiting tyrosine hydroxylase activity, but they mainly utilize local dopamine produced elsewhere by neighboring cells [95,99].

A significant number of bacterial strains produce dopamine and also respond to changes in its peripheral levels. This, in turn, may affect dopaminergic signaling in the central nervous system, transmitted by the afferent vague nerve, either directly or via metabolites such as short-chain fatty acids [66]. Elevated levels of intestinal bacteria producing tyrosine decarboxylase in PD patients may reduce the availability of L-DOPA in the intestines. Their relative abundance is highest in the proximal small intestine, where L-DOPA is the main absorber [66]. The gut microbiota may also modulate host dopamine transporters (DAT), proteins responsible for dopamine reuptake and synaptic clearance in the brain [100,101]. DAT (dopamine transporter) is a presynaptic membrane protein present in dopaminergic terminals that regulates synaptic and extracellular dopamine. It is a key modulator of dopaminergic tone in the central nervous system. The binding of striatal dopamine to DAT (dopamine transporter) in the synaptic cleft of dopaminergic neurons facilitates the reabsorption of dopamine and its subsequent storage in vesicles located within presynaptic terminals. This process is crucial for the regulated release of dopamine at a subsequent stage. It has been established that certain microorganisms, classified within the taxonomic groups *Prevotella*, *Bacteroides*, *Lactobacillus*, *Bifidobacterium*, *Clostridium*, *Enterococcus*, and *Ruminococcus,* have the capacity to modulate receptors, transporters, and specific targets of the dopaminergic pathway, exerting both positive and negative effects [102,103,104,105].

Elevated levels of Bacteroides uniformis bacteria in feces are significantly associated with increased DAT expression, while higher numbers of Prevotella bacteria show a negative correlation [102]. Administration of *Bacteroides uniformis* bacteria via fecal microbiota transplantation increased DAT binding, while *Prevotella copri* was inversely correlated with transporter binding [99]. A large number of Prevotella also leads to an increase in plasma ghrelin concentration, an orexigenic hormone, an intestinal peptide that regulates satiety by acting on the ventral tegmental area (VTA) [106]. Systemically injected ghrelin stimulates motor activity and increases dopamine levels in rodent models [107]. Some species of the genus *Clostridium* (e.g., *Clostridium tetani*) have a dopamine-degrading effect. Metabolites produced by *C. tetani* also inhibit dopamine β-hydroxylase, blocking the conversion of dopamine to noradrenaline [108]. Excess dopamine metabolites lead to toxic accumulation in the cytoplasm, causing oxidative damage as a result of glutathione depletion in the brain. Oxidative stress, in turn, induces apoptosis of dopaminergic neurons and accumulation of α-synuclein protofibrils. Clostridium butyricum, on the other hand, has a beneficial effect on dopamine concentration through the production of significant amounts of short-chain fatty acids (SCFAs), especially butyrate [109], which, by crossing the blood–brain barrier, has a beneficial effect on the function and development of microglia, reduces neuroinflammation, and directly affects dopamine levels in the hypothalamus [71]. *Enterococcus*, belonging to the *Firmicutes phylum,* participates in dopamine production. L-DOPA supplementation enables *Enterococcus faecium* to convert newly introduced L-DOPA into dopamine in the GI tract. Transplantation of both *Enterococcus faecalis* and *Enterococcus faecium* into a mouse model of PD dramatically increased the amount of dopamine in the striatum, as confirmed by dopamine imaging using matrix-assisted laser desorption/ionization mass spectrometry with 2,4,6-trimethylpyranyl tetrafluoroborate (TMP-TFB) (MALDI-MS) [72]. *E. faecalis* also efficiently decarboxylates L-DOPA via pyridoxal phosphate-dependent tyrosine decarboxylase, which is then further metabolized to tyramine. Metabolism of dopamine precursors, i.e., tyrosine and L-DOPA, to tyramine by Enterococcus may prevent the maintenance of sufficient dopamine levels [108]. L-dopa degradation in the gastrointestinal tract is driven primarily by gut microbiota, specifically bacterial tyrosine decarboxylases (TDCs), rather than by host enzymes. This microbial activity is a significant factor in the variable efficacy of L-dopa therapy in Parkinson’s disease. TDCs are produced primarily by lactic acid bacteria (LAB) found in fermented foods and in the human intestine, specifically *Enterococcus faecalis* (formerly *Streptococcus faecalis*), *Enterococcus faecium*, *Enterococcus durans*, and *Lactobacillus brevis*. It is also found in some *Leuconostoc* and *Streptococcus* species [110,111,112]. TDCs can decarboxylate levodopa to dopamine in the peripheral system, thereby limiting its availability in the brain [66,108]. Bacteria carrying TDCs may create a vicious cycle in which peripheral dopamine production affects gut motility, promoting colonization of TDC-producing bacteria [66]

Excess dopamine metabolites lead to toxic accumulation in the cytoplasm, causing oxidative damage as a result of glutathione depletion in the brain. Oxidative stress, in turn, induces apoptosis of dopaminergic neurons and accumulation of α-synuclein protofibrils. Clostridium butyricum, on the other hand, has a beneficial effect on dopamine concentration through the production of significant amounts of short-chain fatty acids (SCFAs), especially butyrate [109], which, by crossing the blood–brain barrier, has a beneficial effect on the function and development of microglia, reduces neuroinflammation, and directly affects dopamine levels in the hypothalamus [71]. Enterococcus, belonging to the Firmicutes phylum, participates in dopamine production. L-DOPA supplementation enables *Enterococcus faecium* to convert newly introduced L-DOPA into dopamine in the GI tract [Transplantation of both *Enterococcus faecalis* and *Enterococcus faecium* into a mouse model of PD dramatically increased the amount of dopamine in the striatum, as confirmed by dopamine imaging using matrix-assisted laser desorption/ionization mass spectrometry with 2,4,6-trimethylpyranyl tetrafluoroborate (TMP-TFB) (MALDI-MS) [104]. *Enterococcus faecalis* also efficiently decarboxylates L-DOPA via pyridoxal phosphate-dependent tyrosine decarboxylase, which is then further metabolized to tyramine. Metabolism of dopamine precursors, i.e., tyrosine and L-DOPA, to tyramine by Enterococcus may prevent the maintenance of sufficient dopamine levels [72]. L-dopa degradation in the gastrointestinal tract is driven primarily by gut microbiota, specifically bacterial tyrosine decarboxylases (TDCs), rather than by host enzymes. This microbial activity is a significant factor in the variable efficacy of L-dopa therapy in Parkinson’s disease. TDCs are produced primarily by lactic acid bacteria (LAB) found in fermented foods and in the human intestine, specifically *Enterococcus faecalis* (formerly *Streptococcus faecalis*), *Enterococcus faecium*, *Enterococcus durans*, and *Lactobacillus brevis*. It is also found in some *Leuconostoc* and *Streptococcus* species [110,111,112]. TDCs can decarboxylate levodopa to dopamine in the peripheral system, thereby limiting its availability in the brain [66,72]. Bacteria carrying TDCs may create a vicious cycle in which peripheral dopamine production affects gut motility, promoting colonization of TDC-producing bacteria [66].

### 5.2. Serotonin and Influence of Gut Microbiota on Serotonin Metabolism

Serotonin, also known as 5-hydroxytryptamine (5-HT), is a neurotransmitter that regulates the secretion of intestinal epithelial cells, intestinal motility, and mucosal immune responses via 5-HT receptors expressed on intestinal epithelial cells, intestinal neurons, and immune cells [113]. It also plays a key role in regulating sleep and wake cycles, nutrition, pain perception, cognitive function, and emotions [74,114]. Approximately 90–95% of serotonin is found in the GI tract, where it is mostly produced and stored by enterochromaffin cells (ECCs) in the intestines. Only 1–2% of serotonin is produced in the CNS [114]. In the central nervous system, 5-HT is synthesized and stored mainly by serotonergic neurons in the brainstem nuclei. These neurons send efferent fibers to, among others, the substantia nigra, striatum, globus pallidus, hypothalamus, thalamus, and cerebral cortex. Additionally, 5-HT fibers exit to the choroid plexus and meninges, where they come into direct contact with cerebrospinal fluid (CSF), potentially reaching other areas of the brain [114].

5-HT is synthesized via the serotonin pathway from an essential exogenous amino acid, tryptophan (TRP). TRP is first converted to 5-hydroxytryptophan (5-HTP), which is then converted to 5-HT by the enzymes tryptophan hydroxylase (TPH) and aromatic amino acid decarboxylase. Both TRP and 5-HTP cross the blood–brain barrier via amino acid transporters and serve as precursors for central 5-HT production, with TPH being the rate-limiting enzyme in this pathway and occurring in two isoforms: TPH1, expressed mainly in peripheral tissues such as the intestine and skin, and TPH2, which is restricted to neuronal cells. After synthesis, 5-HT is further broken down by monoamine oxidase (MAO) to 5-hydroxyindoleacetic acid [115]. Physiologically, only about 1–5% of the TRP consumed enters the serotonin pathway. Peripheral serotonin is unable to cross the BBB, indicating that both central and peripheral serotonin exist in distinct pools. TRP precursors and 5-hydroxytryptophan (5-HTP) cross the BBB as substrates for brain TPH2 to generate functional 5-HT [116,117,118].

TRP metabolism beyond the serotonin pathway occurs via the kynurenine (KP) and indole pathways. KP accounts for approximately 95% of dietary TRP degradation, of which approximately 90% occurs in the liver. Through KP, TRP is converted to kynurenine (KYN) by immune cells, intestinal epithelial cells, or specific gut microbiota through the action of indoleamine-2,3-dioxygenase (IDO) and tryptophan-2,3-dioxygenase (TDO). IDO1 is activated by certain immune and inflammatory stimuli. It is widely expressed in immune and non-immune tissues. IDO2 is found primarily in the liver and kidneys, and its catalytic activity is lower than that of IDO1 [119].

KYN is then metabolized to kynurenic acid (KYNA) by kynurenine aminotransferase (KAT), which is catabolized to anthranilic acid (AA) by kynureninase (KYNU) and converted by kynurenine-3-monooxygenase (KMO) to 3-hydroxykynurenine (3-HK). 3-HK is then converted by KYNU to 3-hydroxyanthranilic acid (3-HAA) [120]. 3-HAA is then catalyzed by 3-hydroxyanthranilic acid 3,4-dioxygenase (3-HAAO) to quinolinic acid (QUIN) and ultimately oxidized nicotinamide adenine dinucleotide (NAD+), necessary to produce adenosine triphosphate (ATP). Physiologically, NAD+ levels decline with age but increase significantly during neuroinflammation. In PD, mitochondrial dysfunction increases NAD+ consumption, driving KP toward the terminal pathway where QUIN is activated to NAD+, further depleting KYN and reducing KYNA synthesis [120,121]. A “dose-dependent effect” is observed with KYNA, with anti-inflammatory activity at low concentrations and an inhibitory effect on glutamatergic synaptic transmission at high concentrations. 3-HAA exhibits anti-inflammatory and neuroprotective effects during inflammation. Kynurenine acid exhibits neuroprotective effects against quinolinic acid-induced excitotoxicity and can modulate extracellular levels of glutamate, dopamine, and GABA [122]. PD patients demonstrate lower serum tryptophan and KYNA concentrations, a lower KYNA/KYN ratio, and higher QUIN/KYNA ratios compared to controls. In patients with advanced PD (Hoehn-Yahr score > 2), KYNA and KYNA/KYN ratios are lower, and QUIN and QUIN/KYNA ratios are even higher compared to patients with early PD. These metabolic changes are independent of dopaminergic drugs, further confirming that KYN metabolism in advanced PD shifts towards the neurotoxic branch/pathway [123]. KP is activated primarily in the liver, and most metabolites originate from peripheral tissues. Some TRP crosses the BBB in its free form via a nonspecific large neutral amino acid transporter and is directly metabolized in the brain. With aging or BBB damage, lipid-soluble substances produced by peripheral metabolism can cross the BBB, affecting KP metabolism in the CNS [124]. Competition between the KP and 5-HT pathways influences TRP allocation. Inflammation- or stress-induced increases in IDO1 expression divert TRP flow away from 5-HT synthesis, resulting in serotonin depletion and accompanying depressive phenotypes. Patients with PD have decreased KYNA levels in the striatum and cerebrospinal fluid and increased QUIN levels in the striatum and cerebral cortex. Higher QUIN levels in the CSF are associated with more severe symptoms and increased excitotoxicity [125]. The KYN/Trp ratio and 3-HK levels are significantly elevated in the putamen nucleus, frontal cortex, and hippocampus of patients with PD. Plasma 3-HK concentrations are closely related to the severity of symptoms and duration of PD. Both QUIN and 3-HK contribute to neuroinflammation and further promote the development of PD through mechanisms such as (activation of) the N-methyl-D-aspartic acid receptor (NMDAR), lipid peroxidation, production of reactive oxygen species (ROS), and increased nitric oxide synthase levels [126].

Elevated 3-HK and QUIN/KYNA ratios suggest excessive activation of the neurotoxic branch, with KMO playing a similar role to IDO1 in this branch. In animal models of PD, increased brain KYNA levels prevent QUIN-induced excitotoxic damage to nigrostriatal dopamine neurons by antagonizing the activation of excitatory glutamate receptors, particularly NMDARs, and activation of the α7 nicotinic acetylcholine receptor (α7nAChR). KAT-I and KAT-II activity is reduced in the plasma of PD patients. In animal models of PD, decreased KAT-I expression in the substantia nigra has been described, leading to decreased KYNA levels [127]. In the colon, tryptophan is directly catabolized and metabolized by gut microbiota via the indole pathway to indoles and their derivatives [128].

Three major pathways of TRP metabolism are directly or indirectly regulated by the gut microbiota. Plasma tryptophan concentrations do not differ significantly between PD patients and their family members under identical dietary conditions. In contrast, indole and IPA levels are significantly elevated in PD patients and correlate with changes in the relative abundance of specific bacterial genera in the gut [128]. This change may be due to structural differences in the gut microbiota of PD patients. IPA has been shown to inhibit neuronal death by reducing endoplasmic reticulum stress and preventing abnormal protein aggregation through its role as a histone deacetylase inhibitor [129]. Gut microbiota may regulate serotonin production by influencing TPH1 in the gut and TPH2 in the brain. Regulation of TPH1 expression is viewed as a rate-limiting step in the biosynthesis of DA, NAd, and adrenaline. 80% of 5-HT is likely produced in the GI tract by *E. coli*, *Hafnia*, Bacteroides, *Streptococcus*, *Bifidobacterium*, *Lactococcus*, *Lactobacillus*, *Morganella*, *Klebsiella*, *Propionibacterium*, *Eubacterium*, *Roseburia*, and *Prevotella* [130].

SCFAs from some microorganisms, such as butyrate and acetate, increase TPH1 activity and 5-HT levels in the gut, as exemplified by Clostridium ramosum [131]. Li et al. confirmed in a study conducted on rats that probiotics such as *Lactobacillus rhamnosus* and *Bifidobacterium longum* showed an increase in serotonin-related enzymes, a decrease in IDO activity, and an improvement in anxiety and depressive symptoms [132]. Microbiota also supports gut development and the formation of enteric neurons via the 5-HT4 receptor. Some gut bacteria, such as lactic acid bacteria and E. coli, can directly produce 5-HT [78]. Some bacterial strains may even carry serotonin transporter-like proteins that uptake serotonin in a manner similar to the human SERT transporter. For example, *Turicibacter sanguinis* utilizes a SERT-like mechanism that can be blocked by SSRIs, leading to increased intestinal 5-HT and changes in microbial composition. These interactions suggest that SSRIs may influence the gut microbiota, and microbial responses may, in turn, influence the efficacy of long-term SSRI treatment. Gut microbiota can influence serotonin (5-HT) levels by modifying serotonin transporter (SERT) expression. Different microbial compositions can increase or decrease SERT expression, leading to corresponding changes in 5-HT levels [85,133].

Gut microorganisms can also alter the expression of serotonin receptors in both the gut and the brain, thereby influencing gut–brain signaling and related behaviors. Specific bacteria, including *Akkermansia muciniphila*, *Lactobacillus plantarum* PS128, *L. paracasei* JY062, *L. gasseri* JM1, and *B. dentium*, have been observed to modulate the activity of key components within the 5-HT system, such as TPH1, SERT, and various 5-HT receptor subtypes. These microbial effects may improve gut motility, modify intestinal secretion, and even reduce anxiety-like behaviors through changes in central 5-HT receptor expression [134,135,136]. Indole is the most abundant tryptophan metabolite in microorganisms, followed by IAA and IPA. Numerous bacterial genera encoding different tryptases produce specific indole derivatives. For example, *Clostridia* produce IAA; *Streptococcus pepticus* produces IA; *Lactobacillus* produces IAld; *Clostridium botulinum*, *Clostridium perfringens*, and *Anaerobacterium sporogenes* produce IPA; *Tridiumsporogenes* and *Ruminococcus* produce tryptamine; *Lactobacillus* and *Bifidobacterium* produce ILA [85,137,138]. More than 85 bacterial species, including *Escherichia coli*, *Bacteroides*, and *Clostridium* spp., produce indole via tryptophanase. Indole, IAld, IPA, and indoxyl-3-sulfate can penetrate the BBB. Furthermore, indole and its derivatives can enter the systemic circulation, where they reduce intestinal permeability and inflammatory responses via pregnancy X receptor (PXR) and AHR-dependent mechanisms [139]. Some indole pathway metabolites also act as AHR agonists on glial cells, modulating CNS inflammation [43]

### 5.3. Acetylcholine and Influence of Gut Microbiota on Acetylcholine Metabolism

ACh is a cholinergic neurotransmitter, a local signaling molecule in the central and peripheral nervous systems. Cholinergic centers are located in the basal forebrain and brainstem. These include the pedunculopontine nuclei, the interpeduncular nuclei, the basal nucleus of Meynert, and the medial septal area and Broca’s diagonal band. In the central nervous system, ACh is essential for many cognitive functions, such as learning, memory, arousal, and attention, as well as for the regulation of sleep and wakefulness [140]. In spinal cholinergic motor neurons, it is responsible for the transmission of nerve impulses at the neuromuscular junction [141]. It is widely distributed in cardiomyocytes, the GI tract, urothelial cells of the urinary bladder, and numerous human leukemic cells [142,143]. In smooth muscle, acetylcholine regulates contraction of the bronchi, intestines, and urinary bladder. It also indirectly modulates glandular secretory function. ACh affects the circulatory system and plays a key role in the autonomic GI system, controlling and regulating its functions [143,144]. ACh, a non-neuronal signaling molecule found in various cell types that express the choline acetyltransferase enzyme (acetyl-CoA, choline O-acetyltransferase, ChAT), may contribute to the regulation of a vast number of cellular functions. Non-neuronal acetylcholine may be a mediator in the development of inflammatory bowel disease and human colorectal cancer [145].

The process of biosynthesis of Ach in the perikaryon of cholinergic neurons from its precursor molecules, choline and acetyl-coenzyme A, is catalyzed by the enzyme choline acetyltransferase (acetyl-CoA, choline O-acetyltransferase, ChAT) in the cytoplasm of cholinergic neurons. Following this, the enzyme is transported to synaptic vesicles via the vesicular acetylcholine transporter (VAChT). ACh is stored in specialized neurosecretory vesicles of neuronal cells and released by exocytosis in specific synaptic clefts [146]. The inactivation of acetylcholine is catalyzed by the enzyme acetylcholinesterase. The process of acetylcholine degradation is extremely rapid (approximately 1.6 × 10^5^ to 8.3 × 10^5^ molecules per minute) [147]. The resultant choline is then reabsorbed from the synaptic cleft and transported back to the presynaptic cell. There, it is acetylated. This allows choline to be reused as a substrate for acetylcholine production. ACh is also produced in non-neuronal cells by the enzyme carnitine O-acetyltransferase (CarAT). Released ACh binds to post- and presynaptic receptors in the synaptic cleft, where it serves a paracrine function. Its action is inhibited/inactivated by acetylcholinesterase (AChE; EC 3.1.1.7) and butyrylcholinesterase (BChE; EC 3.1.1.7) [148].

Choline, a key precursor for ACh synthesis, is obtained from food or as a metabolite of intestinal microorganisms. Several bacterial species, such as *Lactobacillus plantarum*, *Bacillus subtilis*, *Enterobacter*, *Klebsiella*, *Escherichia coli*, and *Staphylococcus aureus*, are implicated in the process of converting choline to ACh. Choline is also converted to trimethylamine (TMA) via the activity of TMA choline lyase [149].

Trimethylamine is a metabolite of the intestinal microbiota, formed as a result of the metabolism of not only choline but also phosphatidylcholine, L-carnitine, and betaine [150]. Furthermore, it is a precursor of trimethylamine N-oxide (TMAO), a low molecular weight uremic toxin in water that significantly increases the risk of cardiovascular events. The source of TMAO in the body is the oxidation of trimethylamine (TMA) by flavin-containing monooxygenase (FMO) in the liver [151,152]. Under physiological conditions, Ach is unable to cross the blood–brain barrier. However, choline from peripheral sources can enter the brain via transporters on capillary endothelial cells [152].

ACh acts through two main types of cholinergic receptors (AChRs): nicotinic (nAChRs) and muscarinic (mAChRs). They are found in the neuromuscular junctions of skeletal muscles, in the autonomic nervous system in sympathetic and parasympathetic ganglia (synaptic transmission), and in the CNS. They exhibit a physiological response to nicotine, acting as ionotropic pentameric receptors (composed of two α subunits and β, γ, and δ subunits). Their activation causes the opening of ion channels for sodium and potassium cations, leading to cell membrane depolarization and cell excitation [153]. Expression of these subunits has been documented in Purkinje cells, some granule cells, astrocytes, and several neurons within the dentate nucleus. mAChRs are responsive to muscarinic hormones and function as metabotropic receptors. These receptors consist of a seven-component G protein-coupled transmembrane receptor (GPCR). Five mAChR subtypes are known, ranging from M1 to M5 (also known as CHRM1–5; M1R–M3R). mAChRs are abundant throughout the body. They are commonly found in the hippocampus, cerebral cortex, thalamus, gastric and salivary glands, smooth muscle, and cardiac tissue [153]. ACh activates enteric neurons and glial cells via M3 and M5 mAChRs, modulating mucosal function, secretion, local blood flow, motility, and digestion [154]. mAChRs prevent IL-1β-induced barrier dysfunction by inhibiting the phosphorylation of myosin L-chain (MLC) by MLC kinase during endocytosis of tight junction factors, including occludin molecules. Non-neuronal ACh derived from the enteric innervation acts on mAChRs in goblet cells, inducing mucus secretion [155].

### 5.4. GABA and Influence of Gut Microbiota on GABA Metabolism

GABA is the primary inhibitory neurotransmitter in the central nervous system. It plays an important role in cognitive processes and responses to stress and pain. It also plays a significant role in behavioral and behavioral disorders [156]. While the role of GABA in the neuropathology of PD remains unclear, it is known that the GABA system plays a critical role in regulating inhibitory tone in the globus pallidus (GP), substantia nigra pars compacta (SNpc), and thalamus, thereby preventing overstimulation of the cerebral cortex. Degeneration of dopaminergic neurons is associated with decreased GABA levels in PD. Dopamine is co-released with GABA by dopaminergic neurons independently of vesicular GABA transporters. GABA release also requires activation of vesicular monoamine transporter 2 (VMAT2), which is also a dopamine neurotransmitter [157]. Furthermore, dopaminergic neurons in the SNpc inhibit the striatum through presynaptic activation of GABA receptors. Dopaminergic neurons acquire GABA through presynaptic uptake and then release it along with dopamine via GABA transporters [158]. Increasing striatal input due to a deficiency of GABA leads to the development of bradykinesia in PD [158]. The GABA system is dysregulated in the neuropathology of PD through disruption of cellular Ca^2+^ signaling. GABA/Ca^2+^ maintains neuronal activity in the central nervous system by preventing intracellular deposition of proteins, Ca^2+^, and Lewy bodies [159]. Imbalanced mitochondrial Ca^2+^ homeostasis is pathogenically linked to neurodegeneration occurring in PD [160]. The organelles primarily involved in Ca^2+^ buffering are the endoplasmic reticulum (ER) and mitochondria. Interactions between lysosomes and ER compartments can generate significant global Ca^2+^ signals within the cell [161]. Elevated mitochondrial Ca^2+^ levels can trigger the opening of the permeability transition pore and initiate apoptosis, as well as directly promote α-syn aggregation [162].

GABA is the primary inhibitory neurotransmitter in the central nervous system. It plays an important role in cognitive processes and responses to stress and pain. It also plays a significant role in behavioral and behavioral disorders [156]. While the role of GABA in the neuropathology of PD remains unclear, it is known that the GABA system plays a critical role in regulating inhibitory tone in the globus pallidus (GP), substantia nigra pars compacta (SNpc), and thalamus, thereby preventing overstimulation of the cerebral cortex [157]. Degeneration of dopaminergic neurons is associated with decreased GABA levels in PD. Dopamine is co-released with GABA by dopaminergic neurons independently of vesicular GABA transporters. GABA release also requires activation of vesicular monoamine transporter 2 (VMAT2), which is also a dopamine neurotransmitter [157]. Furthermore, dopaminergic neurons in the SNpc inhibit the striatum through presynaptic activation of GABA receptors. Dopaminergic neurons acquire GABA through presynaptic uptake and then release it along with dopamine via GABA transporters [158]. Increasing striatal input due to a deficiency of GABA leads to the development of bradykinesia in PD [158].

The GABA system is dysregulated in the neuropathology of PD through disruption of cellular Ca^2+^ signaling. GABA/ Ca^2+^ maintains neuronal activity in the central nervous system by preventing intracellular deposition of proteins, Ca^2+^, and Lewy bodies [159,163]. Imbalanced mitochondrial Ca^2+^ homeostasis is pathogenically linked to neurodegeneration occurring in PD [160]. The organelles primarily involved in Ca^2+^ buffering are the endoplasmic reticulum (ER) and mitochondria. Interactions between lysosomes and ER compartments can generate significant global Ca^2+^ signals within the cell [161]. Elevated mitochondrial Ca^2+^ levels can trigger the opening of the permeability transition pore and initiate apoptosis, as well as directly promote α-syn aggregation [162]. Hyperpolarization of GABA neurons regulates presynaptic neurotransmission and prevents neuronal hyperexcitability by maintaining Ca^2+^ homeostasis [164]. This effect attenuates Ca^2+^ dyshomeostasis-induced dopaminergic neuron damage. Voltage-gated Ca^2+^ channels enhance α-synuclein release in vitro and in vivo, leading to aggregation and the development of synucleinopathy [160,165]. GABA is also a significant neurotransmitter involved in the homeostasis and enteric nervous system (ENS) in disorders such as acid secretion, gastric emptying, gut motility, and pain sensation [165]. There are three main types of GABA receptors: GABA-A and GABA-C (both ionotropic, opening chloride channels and inhibiting neurons) and GABA-B (metabotropic, G protein-coupled, regulating potassium and calcium channels) [166]. Within the central nervous system, GABA is synthesized by GABA neurons and astrocytes. The extent of knowledge regarding the GABA system in the peripheral nervous system is, at present, relatively limited, particularly with regard to the enteric nervous system. It has been demonstrated through a range of studies that both ionic (GABA-A and GABA-C) and metabolic (GABA-B) GABA receptors are present in both neural and non-neural cells of the GI tract [167]. GABA-A receptor mRNA levels were expressed in intermuscular and submucosal neurons and intestinal epithelial cells. The sources of GABA in the intestine have been identified as GABA synthase-containing neurons and endocrine cells of the mucosa. This finding indicates that GABA functions not only as a neurotransmitter but also as an endocrine factor in the GI tract [168]. The substrate for GABA biosynthesis is glutamate, which is decarboxylated by glutamic acid decarboxylase (GAD). GAD is found exclusively in the cytoplasm of GABA terminals. The cofactor for GAD is pyridoxal phosphate. GABA metabolism is initiated by GABA transaminase. The transamination product is succinic semialdehyde (SSA), which is metabolized by SSA dehydrogenase to succinate or alternatively reduced to γ-hydroxybutyrate [169]. Within the GI tract, a specific subset of neurons and endocrine cells located in the mucosa has been identified as the site of synthesis, uptake, and secretion of GABA. These cells play a crucial regulatory role in various aspects of GI function, including motility, secretion, and inflammation [168]. GABA in the CNS is predominantly synthesized via the GABA shunt branch, in which glutamate is decarboxylated by GAD and subsequently metabolized to succinate, which enters the tricarboxylic acid (TCA) cycle. The polyamine degradation pathway provides an additional source of GABA through the conversion of putrescine and related metabolites. Although similar metabolic pathways operate in the gut, GABA synthesis is less efficient due to its dependence on glutamate uptake by transporters and proton-consuming decarboxylation followed by export via GABA antitransporters [168].

Despite the largely impermeable BBB to GABA and glutamate, exogenous GABA can indirectly modulate CNS function via enterocyte-dependent signaling pathways and also act locally on the enteric nervous system or vagus nerve. However, similar to the glutamate synthesis pathway in the CNS, metabolites of carbohydrate fermentation in the large intestine, such as acetate, can cross the BBB and be incorporated into the GABA metabolic cycle. Acetate is transported across the BBB to the hypothalamus and enters the neuroglial GABA circulation pathways. Furthermore, gut-derived GABA, unlike catecholamines, reaches the central nervous system via specific GABA transporters expressed in the BBB [170].

Microbes can import glutamate, export GABA, and in some cases, additionally metabolise GABA via the GABA shunt, which provides an important nitrogen source. The glutamate/GABA antiporter system—primarily GadC and GadT—is crucial for acid resistance, enabling glutamate uptake and GABA efflux [171]. Some bacteria, such as Escherichia coli, can utilize GABA as a sole carbon and nitrogen source and, in acidic environments, utilize GadC to transport glutamate and GABA. Others, such as Bacillus subtilis, lack GAD enzymes for GABA synthesis but can still import GABA extracellularly via permeases homologous to those in *E. coli* [172]. Gut microbiota can modulate the central expression of glutamate and GABA receptors, thereby influencing emotional and behavioral outcomes. The microbiota of individuals with alcohol use disorder reduces mGluR1 and PKCε levels in key brain regions of recipient mice and induces anxiety- and depression-like behaviors [173].

Prebiotics and specific probiotic strains (e.g., *Lactobacillus rhamnosus* HN001, *L. rhamnosus* JB-1, *Bifidobacterium longum*) alter the expression of NMDA and GABA_A/GABA_B receptor subunits in a region-specific manner, often eliciting anxiolytic or antidepressant effects. Microbial dysbiosis in early life reduces the number of α5 and δ GABA_A receptor subunits in the hippocampus, while probiotic supplementation can restore their expression [172]. Perturbations in host glutamate signaling also alter microbial composition. Mice lacking metabotropic glutamate receptors exhibit reduced numbers of Erysipelotrichaceae and *Allobaculum,* whereas Foxo3a^‒/‒^ IL-10^‒/‒^ mice exhibit elevated serum glutamate levels and increased numbers of *Actinobacteria*, *Proteobacteria*, and *Enterobacteriaceae*. Collectively, these results indicate a bidirectional relationship between gut microbiota and the GABA system with implications for mood disorders, models of neuropsychiatric disease, and intestinal inflammation [173,174].

## 6. Therapeutic Interventions in Parkinson’s Disease Potentially Affecting the Gut Microbiota

At present, therapeutic strategies for PD are predominantly symptomatic, aimed at alleviating clinical manifestations and improving patient quality of life. L-DOPA remains the gold standard treatment, compensating for dopamine deficiency in the brain and activating dopaminergic receptors. Increasing evidence indicates a strong association between gut microbiota alterations and PD pathophysiology, suggesting that microbiome-targeted interventions may represent a promising avenue for research and clinical application [175], reducing not only GI symptoms but also playing a key role in the progression of neuroinflammation and neurodegeneration.

### 6.1. Probiotics

Probiotics are defined as live, non-pathogenic microorganisms that provide health benefits when consumed in adequate amounts [176]. The most commonly used probiotics include *Bifidobacterium*, *Lactobacillus*, *Bacillus*, *Streptococcus*, *Enterococcus*, and *Saccharomyces*, as well as combinations of multiple strains [177]. These organisms can be supplied through dietary sources such as fermented foods or through supplements, pharmaceutical preparations, and specialized nutritional products. Probiotics exert their beneficial effects through various mechanisms: they regulate the intestinal microbial population and reduce pathogen colonization and invasion; they increase epithelial cell proliferation and differentiation, strengthening the intestinal barrier, and reducing immunomodulation. Probiotics also produce SCFAs with anti-inflammatory and neuroprotective effects, which penetrate the circulatory system and cross the blood–brain barrier (BBB). They also modulate the activity of central nervous system immune cells, inflammatory cytokines, blood–brain barrier (BBB) integrity, and neurogenesis [178]. They stimulate the synthesis and release of neurotransmitters, influencing BDNF levels, synaptic plasticity, and neuronal function in NDD [178]. In the context of PD, probiotics are attracting increasing research interest not only due to their potential neuroprotective properties and modulation of the GBA by influencing the composition of the gut microbiota, inhibiting the growth of pathogenic bacteria, reducing inflammation, attenuating oxidative stress, and improving neurotrophic and metabolic support, but also due to the reduction of disease symptoms [121]. Lu CS et al. [179] conducted an open-label, single-arm, controlled study. Twenty-five PD patients took 60 billion colony-forming units of PS128 once nightly for 12 weeks and achieved significant improvement in the UPDRS-III in both the OFF and ON states (*p* = 0.004 and *p* = 0.007, respectively). Furthermore, the PS128 intervention significantly prolonged the duration of the ON and OFF states and improved the Parkinson’s Disease Questionnaire-39 (PDQ-39) scores. However, no clear effect of PS128 on non-motor symptoms was observed. Sun H et al. [180] investigated the effect of co-administration of probiotics and levodopa with benserazide and dopamine agonists in patients with PD in a 3-month randomized, double-blind, placebo-controlled clinical trial. Forty-five patients received Bifidobacterium animalis subsp. lactis Probio-M8 (Probio-M8) and 29 received a placebo. Co-administration of Probio-M8 with dopaminergic medications resulted in additional benefits, including improved sleep quality, reduced anxiety, and reduced gastrointestinal symptoms. Furthermore, metagenomic analysis revealed significantly more species-specific genomic codes (SGBs) of *Bifidobacterium animalis*, *Ruminococcaceae*, and *Lachnospira*, and significantly fewer *Lactobacillus* fermentum and *Klebsiella oxytoca* in the Probio-M8 group (*p* < 0.05). *Lactobacillus* fermentum correlated positively with the results of the UPDRS-III, Hamilton Anxiety Rating Scale (HAM-A), Hamilton Depression Rating Scale (HAMD-17), and negatively with the Mini-Mental State Examination (MMSE). *Klebsiella oxytoca* correlated negatively with stool hardness. Co-administration of Probio-M8 increased the level of SGBs involved in the degradation of tryptophan, gamma-aminobutyric acid, short-chain fatty acids, and the biosynthesis of secondary metabolites. Magistrelli L et al. [181] randomly assigned 40 PD patients receiving stable antiparkinsonian therapy to groups receiving active probiotics (*Bifidobacterium animalis* subsp. lactis BS01 LMG P-21384, *Bifidobacterium longum* BL03 DSM 16603, *Bifidobacterium adolescentis* BA02 DSM 18351, fructooligosaccharides and maltodextrin–group A) or placebo (maltodextrin–group B). After 12 weeks, the probiotic-treated group observed significant improvement in the Unified Parkinson’s Disease Rating Scale (UPDRS)-III (*p* = 0.028) and non-motor symptoms of Non-Motor Symptoms Scale (NMSS) (*p* = 0.041) and significant improvement in the gastrointestinal subcomponent of gastroenteritis (*p* = 0.021). Both groups showed a decrease in interferon-γ levels, but the probiotic group also showed a significant decrease in interleukin-6 and a slight increase in transforming growth factor beta (TGF-β). To evaluate the effect of probiotic supplementation on the expression of genes related to inflammation, insulin, and lipids, Borzabadi S et al. [182] conducted a randomized, double-blind, placebo-controlled pilot study in which 25 PD patients took probiotic supplements at a dose of 8 × 109 CFU/day, and 25 PD patients received a placebo. After 12 weeks of intervention, probiotic intake decreased the gene expression of interleukin-1 (*p* = 0.03), interleukin-8 (*p* < 0.001), and tumor necrosis factor-alpha (*p* = 0.04), and increased the expression of transforming growth factor-beta (*p* = 0.02) and peroxisome proliferator-activated receptor-gamma (*p* = 0.03) compared to placebo in peripheral blood mononuclear cells (PBMCs). Probiotic supplementation did not affect low-density lipoprotein receptor (LDLR) or vascular endothelial growth factor (VEGF) gene expression in PBMCs, nor did it affect biomarkers of inflammation and oxidative stress. Zali A et al. [183] investigated the effect of co-administration of probiotics and vitamin D on various inflammatory parameters in PD patients who were randomly assigned to one of two treatment groups: probiotic/vitamin D supplements (*n* = 23) and placebo capsules (*n* = 23) for 12 weeks. Consumption of probiotic/vitamin D supplements led to a significant reduction in the levels of interleukin-1 beta, interferon-gamma, interleukin-6, and malondialdehyde, with a concomitant increase in interleukin-10 and total antioxidant capacity (TAC) compared to the placebo group, and to a significant reduction in disease severity, anxiety, and gastrointestinal problems compared to the placebo group. Lenta V et al. [184] conducted a multicenter, randomized, double-blind, placebo-controlled trial (NCT05146921) using probiotics (35 participants) for 12 weeks. An increase in the number of bacteria with beneficial health-promoting properties (*Odoribacteraceae*, *Enterococcaceae*, and *Blautia faecicola*) was observed in the active group compared with placebo (*p* ≤ 0.05) (primary endpoint). Plasma levels of the proinflammatory cytokine TNF-α decreased in the probiotic group and increased in the placebo group (*p* < 0.05) (no changes in SCFA levels were observed), and the duration of the off-state was shortened, and Non-Motor Symptoms Scale (NMSS) scores were reduced (*p* < 0.05) in the active group (secondary/exploratory endpoints).

### 6.2. Prebiotics

Prebiotics are defined as dietary components non-digestible in the upper human GI tract that are selectively metabolized by beneficial gut microorganisms in the host’s large intestine, thereby providing health benefits [185]. Prebiotics have been shown to provide an energy source for specific gut bacteria, thereby altering their composition and activity. Fermentation is a complex series of processes with the resultant by-products promoting cross-feeding between microorganisms. The acidic fermentation products produced by prebiotic metabolism have been shown to lower intestinal pH, thereby affecting the composition and abundance of gut microbiota. This, in turn, has been demonstrated to provide a favorable environment for beneficial bacteria, such as Lactobacilli and Bifidobacteria, whilst simultaneously inhibiting the growth of pathogenic bacteria [186]. In contrast to probiotics, prebiotics do not contain viable microorganisms; they consist primarily of fermentable dietary fiber. Despite the limited number of clinical studies examining the relationship between prebiotics and PD, existing evidence suggests that prebiotics can modulate the immune response, improve gut motility, relieve constipation, and improve overall GI function [121]. Prebiotics are primarily derived from natural food sources, although several synthetic forms exist, including inulin, galactooligosaccharides (GOSs), fructooligosaccharides (FOSs), and SCFAs [187]. Studies in animal models have shown that prebiotics increase the number of beneficial commensal bacteria (*Bifidobacteria*/*Lactobacilli*), SCFA production (especially butyrate), improve antioxidant enzyme activity, and reduce markers of oxidative stress and inflammation [185,186,187,188,189,190]. Synbiotic approaches may offer additional benefits by combining substrate delivery with probiotic colonization [187]. Several small clinical trials have directly assessed the effect of prebiotic supplementation on the gut microbiota and clinical symptoms of PD patients. Hall DA et al. [191] in an open-label, non-randomized study of newly diagnosed, untreated (*n* = 10) and treated participants with PD (*n* = 10) assessed the effect of a 10-day prebiotic intervention. The results indicated that the prebiotic intervention was well tolerated (primary outcome) and safe (secondary outcome) in participants with PD. In contrast, a prospective, randomized, double-blind study by Hegelmaier [192], in which 72 individuals with PD received 6 months of supplementation with propionic acid (postbiotic) and butyric acid (postbiotic) and/or the prebiotic fiber 2’-fucosyllactose, demonstrated significant improvement in motor symptoms in both intervention groups. These motor benefits were accompanied by a clinically significant reduction in levodopa doses. Becker A et al. [193], in an interventional, monocentric, open-label clinical trial, investigated the effect of resistant starch on dietary habits, short-chain fatty acids, and gut microbiota in PD (RESISTA-PD; ID: NCT02784145). The aim of this study was to alter fecal SCFA concentrations through an 8-week prebiotic intervention with resistant starch (RS). Thirty-two PD patients received RS (PD + RS), 30 controls received RS, and 25 PD patients received dietary advice only. In the PD + RS group, fecal butyrate concentrations significantly increased, and fecal calprotectin concentrations significantly decreased after 8 weeks of RS intervention. Clinically, a reduction in the burden of non-motor symptoms was observed in the PD + RS group. Marichella M et al. [194] conducted a tertiary, randomized, double-blind, placebo-controlled study in patients with PD with constipation confirmed by the Rome III study, based on data from 2-week stool diaries at baseline. One hundred and twenty patients were assigned to receive fermented milk containing multiple probiotic strains and prebiotic fiber or a placebo once daily for 4 weeks. For the primary endpoint, consumption of fermented milk containing probiotics and prebiotics resulted in a greater increase in the number of complete bowel movements (CBM) than placebo (*p* = 0.002). For key secondary endpoints, more patients in the probiotics and prebiotics group than placebo reported 3 or more complete bowel movements (*p* = 0.030) and an increase of one or more complete bowel movements (*p* = 0.004) at weeks 3 and 4.

### 6.3. Synbiotics

Synbiotics, defined as synergistic formulations combining probiotics with prebiotics, enhance the viability and functional activity of probiotic strains by selectively increasing their colonization in the gastrointestinal tract, inhibiting the proliferation and metabolic activity of pathogenic microorganisms, modulating the host immune response, and restoring gut microecological homeostasis while exerting significant therapeutic benefits [195]. Although research on interventions on the gut microbiota in PD has gained momentum, evidence specific to synbiotics is still relatively limited. Most studies to date have been conducted in animal models. Li C et al. [196] demonstrated that the use of synbiotics is more effective compared to separate administration of probiotics or prebiotics alone by administering the probiotics *Lactobacillus rhamnosus* AS 1.2466 and *Lactobacillus delbrueckii* ssp. bulgaricus ATCC 11842 to mice, along with the prebiotics xylooligosaccharides and red ginseng extracts. The probiotics and prebiotics rapidly reduced the Firmicutes/Bacteroidetes ratio, inhibiting the growth of harmful bacteria (*Klebsiella* and *Escherichia coli*) and accelerating the regeneration of beneficial intestinal microorganisms (such as Lactobacillus). Liu X et al. [163] reported that a symbiotic containing the prebiotic polymannuronic acid (PM) and the probiotic *Lacticas-eibacillus rhamnosus* GG (LGG) demonstrated its neuroprotective properties in relation to PD. The combination of PM and LGG showed significantly better neuroprotective effects than PM or LGG alone and improved movement, activity, muscle strength, and increased tyrosine hydroxylase gene/protein expression in the midbrain and striatum of PD mice. Only one human study—a single-arm, open-label study—has tested a defined synbiotic: Enterolactis Duo, containing the probiotic strain *Lacticaseibacillus paracasei* DG and the prebiotic fiber inulin [197]. Thirty patients with stable PD who met the diagnostic criteria for functional constipation and/or irritable bowel syndrome with constipation, according to the Rome IV Criteria, were enrolled and given Enterolactis Duo for 12 weeks. 16S rRNA gene profiling and SCFA quantification were performed to characterize the microbial ecosystem of stool samples collected before (*n* = 22) and after (*n* = 9) synbiotic administration. After treatment, patients achieved better scores on the Movement Disorder Society Unified Parkinson’s Disease Rating Scale (MDS-UPDRS), Scales for Outcomes in Parkinson’s disease-Autonomic Dysfunction (SCOPA-AUT), Toronto Alexithymia Scale-20 (TAS-20), and Hamilton Depression Rating Scale (HAM-D). Gastroenterological tests showed improvement in the Patient Assessment of Constipation-Symptoms (PAC-SYM) score, the number of total bowel movements, and the Bristol Stool Form Scale (BSFS).

### 6.4. Postbiotics

Postbiotics are a new category of bioactive compounds defined by the International Scientific Association for Probiotics and Prebiotics (ISAPP) as “preparations of non-living microorganisms and/or their components that confer a health benefit to the host” [198]. Postbiotics are formed as a result of probiotic metabolism and include: short-chain fatty acids (SCFAs), extracellular polysaccharides, peptides, cell wall fragments, and other cellular components [199]. Postbiotics are considered safer than probiotics because they do not require the presence of living microorganisms; therefore, the risk of interaction between inactivated microorganisms and the host microbiota is eliminated [200]. These compounds can effectively modulate the immune response and demonstrate anti-inflammatory and protective effects on the integrity of the intestinal epithelium [201,202]. The ability to regulate the immune response, strengthen the intestinal barrier, and modify the gastrointestinal microbiota may have a beneficial effect on neuropsychiatric conditions via the gut–brain axis [203,204]. Postbiotic preparations most often contain Gram-positive bacteria from the genera *Lactobacillus* and *Bifidobacterium*, which contain SCFAs, peptides, and exopolysaccharides, which contribute to their anti-inflammatory properties. Postbiotics derived from Gram-negative bacteria such as *Akkermansia muciniphila* are used less frequently. They contain membrane components—lipopolysaccharides (LPS)—that exhibit immunoregulatory properties [200,205]. The use of postbiotics as agents supporting CNS homeostasis is a relatively new topic.

Few studies have investigated postbiotic metabolites potentially affecting GBA in PD patients. Avagliano C et al. [206] described a two-stage mouse model of PD involving ceftriaxone (CFX)-induced dysbiosis followed by intestinal inflammation prior to intravenous injection of 6-hydroxydopamine (6-OHDA), in which a beneficial effect of postbiotic sodium butyrate was noted by simultaneously reducing motor deficits, neuroinflammation, and colonic damage, as well as by altering the composition of the gut microbiota. Bohnen JLB et al. [207] assessed the safety and potential biomechanistic effects of tributyrin (a prodrug of butyrate) in an open-label clinical trial. Fourteen PD patients and three control subjects completed a 30-day (±7 days) intervention consisting of dietary supplementation with tributyrin (500 mg taken orally three times daily), demonstrating a satisfactory safety profile and high compliance. Nine patients completed a PET scan with [11C] butyrate before and after the intervention, which allowed for the assessment of treatment-related changes in butyrate uptake by the brain, liver, heart, and gastrointestinal tract, confirming involvement in the target process, i.e., changes in butyrate availability in individual organs and systemic anti-inflammatory effects, as well as improved cognitive and motor functions. Other studies focused on assessing the effect of this compound on cognitive, sleep, and emotional disorders, as well as stress response in healthy individuals [208]. Among other findings, improved platelet mitochondrial activity, measured by cytochrome oxidase (COX) activity, was observed in Alzheimer’s disease patients after administration of S-equol [209]. Short-chain fatty acids delivered to the colon have also been reported to attenuate the cortisol response to psychosocial stress in healthy men [210].

### 6.5. Antibiotic Therapy

Another way to modify the GBA is antibiotic therapy, which appears to have the greatest impact on changes in the gut microbiota. Antibiotics are primarily used to prevent bacterial infections; however, in neurodegenerative diseases, interventions are increasingly being conducted to investigate their potential anti-inflammatory, immunomodulatory, antioxidant, and neuroprotective properties [211,212,213]. In the case of PD, it is also postulated that the administration of antibiotics for specific dysbiotic conditions can alleviate not only gastrointestinal symptoms but also motor symptoms [212,213]. Rifaximin, an antibiotic belonging to the rifamycin group, used to treat intestinal infections and not penetrating the intestinal-blood barrier, has been tested both in animal models and in a few clinical studies. Hong et al. [214] analyzed the effect of long-term rifaximin treatment in transgenic PD mice and the effect of seven days of rifaximin treatment in PD patients. Rifaximin treatment elicited a substantial shift in the composition of the gut microbiota in transgenic PD mice. Specifically, it led to a decrease in the relative abundance of Prevotellaceae UCG-001 and an increase in the relative abundance of *Bacteroides*, *Muribaculum*, and *Lachnospiraceae* UCG-001. The modification of the microbiome of PD mice was associated with decreased serum levels of proinflammatory cytokines, such as IL-1β, IL-6, and TNF-α, claudin-5, and occludin, suggesting reduced inflammation and protection of BBB integrity. Mice treated with rifaximin demonstrated better motor performance and memory than control mice, as well as lower microglial activation and increased neuronal survival in the hippocampus. In contrast, in PD patients, rifaximin treatment resulted in an increased relative abundance of Flavonifractor bacteria 6 months after treatment. Furthermore, the change in plasma levels of proinflammatory cytokines was negatively correlated with baseline plasma levels of interleukin-1α. Conversely, the eradication of small intestinal bacterial overgrowth (SIBO) in PD patients with rifaximin, as reported by Fasano A et al., resulted in an improvement in motor fluctuations without affecting the pharmacokinetics of levodopa. Moreover, the recurrence rate of SIBO after six months was 43% [215]. Research conducted in the Drosophila DJ-1A PD model has demonstrated that monocycline, through its antioxidant and anti-inflammatory properties, may exert a protective effect on the dopaminergic system [216]. According to Radad K et al., minocycline may also attenuate the rotenone-induced progressive loss of tyrosine hydroxylase-immunoreactive neurons in rats [217]. However, a randomized, double-blind phase 2 study involving 195 PD patients conducted in the United States and Canada did not demonstrate significant benefits of this antibiotic [218]. Cui et al. [219] reported that vancomycin administered to MPTP-induced PD mice inhibited inflammation via the TLR4/MyD88/NF-κB/TNF-α signaling pathway, while simultaneously increasing *Akkermansia* and *Blautia* levels and reducing astrocyte and microglial activation. Vancomycin also resulted in a reduction in motor symptoms. Research conducted in murine models of MPTP-induced PD has demonstrated that treatment with ceftriaxone, a third-generation cephalosporin, results in the suppression of Proteus species while concomitantly fostering the proliferation of *Akkermansia* species [219]. Furthermore, ceftriaxone reduces the levels of key proinflammatory mediators: TLR-4, NF-κB, and MyD88 in the colon; MyD88, TLR-4, IL-1β, TNF-α, and NF-κB in the brain; and IL-1β, TNF-α, and IL-6 in serum [205,220,221]. Conversely, there is evidence that ceftriaxone-induced dysbiosis exacerbates motor symptoms in 6-OHDA-treated mice and correlates with dopaminergic neuron toxicity, as well as intestinal and systemic inflammation [222]. Lazzarini M et al. showed that doxycycline can block 6-hydroxydopamine (6-OHDA)-induced neurotoxicity in mice by inhibiting the expression of microglia and astrocytes [223] and can inhibit LPS-induced dopaminergic neuron degeneration by reducing the expression of microglial histocompatibility complex II (MHC II) [224]. Doxycycline can also effectively eliminate cognitive deficits and daily activity deficits in A53T mice by reducing the structural stability of pathological α-synuclein and activating striatal glial cells [225]. Similarly, in rats with 6-OHDA-induced PD treated chronically with neomycin, pimaricin, bacitracin, and vancomycin, this antibiotic cocktail was reported to alleviate inflammation, reduce neurotoxicity, and prevent dopaminergic neuron death, which translated clinically into reduced motor symptoms [226]. In mice with MPTP-induced PD, the use of an antibiotic mixture consisting of ampicillin, metronidazole, and neomycin sulfate resulted in the preservation of TH and dopamine transporter immunoreactivity, which typically disappears after MPTP administration. This was associated with an increase in *Proteobacteria* abundance and a simultaneous reduction in *Deferribacteres* and *Saccharibacteria* (TM7) abundance [227].

Antibiotics may also eliminate certain beneficial microbial populations, resulting in gut dysbiosis and neurological dysfunction [228]. A population-based study conducted in Finland on 13,976 people with PD and 40,697 healthy individuals showed that taking certain antibiotics, especially macrolides and lincosamides, correlated with an increased risk of developing PD even 10–15 years after exposure. However, the results of another prospective study involving 59,637 women did not show any correlation between antibiotic use and the incidence of PD [229].

### 6.6. Fecal Microbiota Transplantation

Fecal microbiota transplantation (FMT) involves transplanting functional gut flora from the stool of healthy donors into the patient’s gastrointestinal tract. The goal of this procedure is to increase the number of beneficial bacteria and reduce the population of harmful bacteria, restore intestinal homeostasis, and thus mitigate disease progression [230]. This approach allows for the restoration of dysbiotic gut microbiota and has been approved by organizations such as the WHO and FDA for the treatment of gastrointestinal infections and related conditions. FMT is believed to modulate gut microbiota through immunological, endocrine, metabolic, and neuroregulatory pathways, thereby influencing the symptoms of neurological disorders. Preclinical studies have demonstrated several mechanisms by which FMT beneficially affects gastrointestinal function, alleviates motor symptoms, and delays neurodegeneration in PD [121]. FMT reduces inflammation and oxidative stress, mitigates the neurotoxic effects of microglia and astrocytes, lowers LPS levels in the colon, and reduces the secretion of proinflammatory cytokines while increasing levels of anti-inflammatory factors [58]. FMT modulates inflammatory signaling pathways by enhancing BBB integrity, increasing the expression of tight junction proteins (Zonula Occludens-1, Zonula Occludens-2, Occludin, and Claudin-5) [28,231], and reducing the level of TLR4/TNF-α signaling in gut and brain tissues, resulting in protection of dopaminergic neurons with increased levels of dopamine (DA) and 5-HT in the striatum [217] and reducing mitochondrial damage and increasing their antioxidant capacity [232,233]. Sampson et al., 2016 [234] demonstrated that transplantation of fecal microbiota from PD patients into mice enhances microglial activation and α-syn aggregation through metabolite modulation (mainly SCFAs), which clinically correlates with the development of motor dysfunction. Subsequent preclinical studies in PD have demonstrated that FMT restores gut microbiota diversity by increasing SCFAs levels (especially butyrate) and reduces pathological α-syn aggregation in the ENS and substantia nigra, ultimately alleviating motor dysfunction [232,235]. Furthermore, it has been observed that FMT can regulate SCFAs levels by increasing the expression of SCFA receptors, thus alleviating pathological symptoms [236].

Clinical trials using FMT in PD patients have demonstrated a significant effect of this potential therapeutic modality on motor and non-motor symptoms. Kuai et al., 2021 [237] reported that FMT significantly reduced Bacteroides while increasing *Prevotella* and *Blautia* populations in patients with constipation, resulting in a significant reduction in constipation and significant improvements in both the Patient Assessment of Constipation Quality of Life (PAC-QOL) and the Wexner scale. In the same year, Segal et al. [238] published the results of colonoscopic infusion FMT in six patients with PD, demonstrating significant improvements in motor, non-motor, and constipation symptoms over a six-month follow-up period. DuPont et al. [239] conducted a 12-week randomized, controlled, double-blind study in 12 patients with mild to moderate PD who were administered oral lyophilized FMT products. The study demonstrated significant improvement in constipation and motility. Improvements were observed in the Nonmotor Symptom Scale (NMS), PDQ-39, Geriatric Depression Scale (GDS), Parkinson’s Anxiety Scale (PAS), and UPDRS, although only the improvement in the UPDRS reached statistical significance. Xue et al. [240] reported significant improvements in the Pittsburgh Sleep Quality Index (PSQI), 39-item PD Questionnaire (PDQ-39), Non-Motor Symptom Questionnaire (NMSQ), Hamilton Anxiety Scale (HAMA), Hamilton Depression Rating Scale (HAMD), and Unified Parkinson’s Disease Rating Scale-III (UPDRS-III) one and three months after FMT and found colonic FMT. They also found colonic FMT to be more effective and safer than nasoenteric FMT, likely due to better colonization of the colonic microbiota. Cheng et al. [241] conducted an FMT study involving 56 participants with mild to moderate PD and observed improved Mini-Mental State Examination (MMSE) scores and reduced MDS-UPDRS total scores after 12 weeks. However, they found no significant changes in scales monitoring symptoms of anxiety and depression. A subsequent GUT-PARFECT study involving 46 participants also demonstrated improved MDS-UPDRS scores in the FMT group compared to the placebo group [242]. Although long-term studies confirming the efficacy of FMT are still lacking, existing evidence indicates its short-term safety. Most adverse events are mild, self-limiting gastrointestinal symptoms such as diarrhea, constipation, flatulence, nausea, vomiting, and abdominal discomfort. It appears that the observed partial therapeutic effect of FMT on motor and some non-motor symptoms of PD may also be attributed to reduced numbers of tyrosine decarboxylase-producing bacteria in the stool and, therefore, increased bioavailability of levodopa [243].

### 6.7. Dietary Intervention

Recent research suggests that modifying diet to influence the gut microbiota may help prevent neurodegenerative diseases and improve their symptoms by promoting a healthier microbial profile and increasing the generation of beneficial metabolites [244]. Diet strongly influences the composition and function of the gut microbiota. Dietary fiber—carbohydrates that cannot be digested by human enzymes—acts as a primary substrate for beneficial microbial species. Commensal bacteria such as *Bifidobacterium* and *Lactobacillus* metabolize fiber through fermentation, producing short-chain fatty acids (SCFAs), which are an energy source for intestinal epithelial cells, support the integrity of the mucosal barrier, and also modulate neural activity within the central nervous system through the GBA [31,245]. Unsaturated fatty acids—particularly omega-3 polyunsaturated fatty acids (PUFAs)—have anti-inflammatory properties and play a regulatory role in shaping the gut microbiota. Omega-3 PUFAs can modulate gut immune responses, suppress the proliferation of pathogenic bacteria, and promote the expansion of beneficial taxa such as *Bifidobacterium* and *Lactobacillus*, thereby contributing to a healthier microbial profile. In addition, omega-3 PUFAs exert direct effects on the brain by participating in neuronal membrane synthesis, enhancing neuronal function, and improving cognition and mood [31,246]. Taking everything into account and comparing to known diets, the Mediterranean diet is an example of a diet that supports beneficial gut microbiota activity and neuroprotection, reduces harmful metabolites, and may lower L-DOPA requirements, helping to improve both motor and non-motor symptoms in PD [175,247]. Fermented foods such as yogurt, kefir, sauerkraut, kimchi, and miso serve as natural sources of probiotics, which can enhance gut microbial diversity and support the maintenance of a stable and balanced intestinal ecosystem [248].

### 6.8. Other Methods

In PD mouse models, acupuncture improves motor function and protects dopaminergic neurons by reducing inflammation, preventing apoptosis, and restoring gut microbiota balance. Additionally, engineered probiotics capable of releasing therapeutic molecules such as Exendin-4 via the GBA represent a promising microbiome-based strategy for modulating brain function [249].

### 6.9. Translational Challenges and Evidence Gaps in Microbiota-Targeted Interventions

Most mechanistic studies and robust results come from animal models; human studies are small, heterogeneous, and short-term. The strongest evidence, confirmed by clinical trials, concerns probiotics. For prebiotics, postbiotics, FMT, and antibiotics, the evidence is weaker, and the studies are very preliminary. Preclinical models of PD exposed to probiotics suggest reduced systemic inflammation, manifested by reduced levels of TNF-α and CRP, and anti-inflammatory changes in cytokine profiles, as well as changes in metabolites and pathways related to neurotransmitters (dopamine, tryptophan, acetylcholine, and GABA). Reductions in neuroinflammation or reduced dopamine loss likely result from neuroprotective effects achieved by modulating the composition of the gut microbiota, increasing beneficial taxa, and activating SCFA-related pathways [250,251,252]. Although animal models of PD show that long-term probiotic use protects dopaminergic neurons in the substantia nigra [233], the effect of probiotic microorganisms on PD progression in humans remains unproven. There is no conclusive evidence that probiotics slow the progression of PD by reducing α-synuclein pathology in humans. Several RCTs [253,254] with low to moderate certainty have shown improvements in constipation, bowel frequency, and stool consistency. Single randomized clinical trials have shown significant reductions in plasma proinflammatory markers in PD patients [184]. The effect on motor function was modest and inconsistent, although several studies [179,180,181] have shown significant improvements in MDS-UPDRS/UPDRS Part III. Non-motor outcomes (depression, anxiety, RBD, quality of life) often improved, but with small sample sizes, short duration, and low GRADE (Grades of Recommendation, Assessment, Development, and Evaluation) scores [184,253,254,255]. Studies conducted in rodent models of PD using human-derived microbiota transfers and defined prebiotic manipulations provide strong causal evidence that gut microbiota composition modulates α-syn pathology and motor deficits in PD models by reducing inflammation and oxidative stress in the peripheral and central systems, modulating cytokines (TNF-α, IL-6), glutathione, and antioxidant pathways [188,256]. In humans, prebiotic studies demonstrate temporal associations between intervention and changes in microbiome and metabolite composition, and between changes in microbiome and metabolite composition and symptom modification. Randomized clinical trials using prebiotics are few, mostly pilot studies of short duration and very small sample sizes, and demonstrate increased SCFA concentrations, a shift towards saccharolytic (beneficial) taxa, reduced inflammation, improvement in intestinal symptoms, and initial improvement in the UPDRS scale [191,257,258]. There is a lack of direct data linking prebiotic-induced microbiome changes to α-syn aggregation in the CNS, nigrostriatal neuron survival, and/or imaging biomarkers. Therefore, at this stage, we can only speak of correlation or biological probability, rather than a proven cause-and-effect relationship modifying the course of the disease.

Postbiotic preparations are primarily discussed as future options rather than as tested interventions for patients with PD. Preclinical heterogeneity, wide variability in PD induction (MPTP, 6-OHDA, rotenone, genetic modification), postbiotic dosing, outcome measures, and often short follow-up periods limit the reliability of studies in animal models of humans with progressive, late-treated Parkinson’s disease. Many rodent studies lack randomization, blinding, or direct verification of the microbiome, increasing the risk of bias and resulting in low reproducibility [259,260]. Single metabolites or defined postbiotic preparations are rarely isolated and causally investigated. Clinical trials are lacking, and RTCs and longitudinal studies are particularly needed. In the case of symbiotic RCTs, clinical trials demonstrate significant heterogeneity resulting from different interventions (probiotics vs. synbiotics vs. FMT vs. antibiotics), doses, duration, symptom severity at baseline, and outcome measures. The only [197] clearly described RCT using synbiotics in humans demonstrated short-term clinical improvement; however, it did not assess biomarkers relevant to disease progression, such as α-Syn, PET imaging, or cerebrospinal fluid markers. However, interventions using genetically modified male mouse models, often administered before or near the temporal induction of PD, are difficult to translate to late-stage human Parkinson’s disease in both sexes, likely overestimating apparent disease-modifying effects. Animal analyses also group synbiotics with other interventions. Animal studies indicate neuroprotection. Refs [163], while human RCTs [197] show modest improvements in motor symptoms and constipation, with low-to-moderate quality evidence, significant heterogeneity, and no definitive evidence of disease modification or causality. Similarly, in the case of FMT, limited longitudinal interventions using young male rodent models and administered before or at a very early stage of disease poorly reflect the progressive, late-treated nature of human PD and contribute to translational failure of disease-modifying therapies [261]. In humans with PD, randomized controlled trials have been conducted with varying donor selection, preparation, routes of administration (upper and lower GI tract), dosage, and outcome sets [221,222,223,224]. This clinical heterogeneity, as well as the short follow-up period, limits the power to detect or compare effects. The association of the microbiome with PD: consistent patterns of dysbiosis, but the lack of a stable, consensus microbial signature and the cross-sectional nature of human studies mean that the direction of effect (PD causing dysbiosis vs. dysbiosis causing PD) remains uncertain [260,262]. The proposed mechanisms–LPS-TLR4/TNF-α/NF-κB modulation, SCFAs, barrier integrity, and neuroinflammation–are well documented in mice [261] but have not yet been mapped to causal biomarkers in humans. In multiple rodent models of PD, antibiotics that alter the gut microbiota have been shown to protect dopaminergic neurons and improve motor deficits and/or increase levodopa bioavailability, often with reduced neuroinflammation. Tetracyclines, cephalosporins, doxycycline, vancomycin, monocycline, and rifamycins have been reported to exert neuroprotective effects in vitro/in vivo, affecting α-syn aggregation, mitochondrial function, and microglia [205,215,217,220,221,222,223,224,225,226,227]. Studies in humans have shown that certain classes of antibiotics are associated with a slightly increased risk of PD with long-term therapy [74]. Reported clinical trials, most of them low- to moderate-powered, vary in design (e.g., case–control, cohort, ecology), exposure settings (prescription records vs. memory; duration vs. number of classes), latency windows, and control for confounding factors, leading to significant heterogeneity [215,228,229,263]. Retrospective observational designs cannot rule out reverse causation (infections associated with prodromal PD) or confounding factors related to infection, healthcare utilization, or comorbidities. Ecological correlations (country-level antibiotic use and PD prevalence) are insufficient for causal inference [228,229,263]. Dose-response relationships, critical time windows, and risk-benefit ratios for specific classes are incompletely characterized. Reports do not provide details on duration and adherence to therapy [228,263]. It is unclear whether the observed associations between PD and antibiotics reflect drug effects or underlying infections in PD. RCTs of antibiotics as disease-modifying drugs in PD are lacking. Direct mechanistic data in humans linking specific antibiotic-induced microbiome changes to incident PD are scarce; most evidence is associative.

## 7. Summary

The formulation of the microbiota–gut–brain axis theory has opened up new avenues of research and led to numerous studies that have broadened our understanding of the pathogenesis, phenotypic heterogeneity and clinical course of PD. Current PD pathogenesis models, which are based on the fundamental Braak’s hypothesis, suggest that a subtype of the disease exists in which pathological changes may originate in the gut many years before brain pathology and motor symptoms appear. Further findings now corroborate Braak’s theory that gut microbiota influence the nervous system’s function by controlling the production and transmission of pathological α-synuclein along the gut–brain axis. Key factors influencing gastrointestinal pathology and the progression of PD include increased intestinal permeability, chronic inflammation, oxidative stress, α-synuclein aggregation, and neurotransmitter imbalances. Neurotransmitters play a vital role in maintaining intestinal homeostasis, including the absorption of nutrients, blood flow, the gut microbiome, the local immune system, and overall gut motility. Accumulating evidence suggests that the gut microbiota can regulate neurotransmitter synthesis and metabolism in both the gut and the brain, and can indirectly stimulate the activity of the enteric central nervous system via the vagus nerve, which receives signaling from the enteric nervous system. Research on the gut–microbiome–brain axis has paved the way for the identification of new therapeutic targets, including symptomatic, disease-modifying, and preventative treatments for PD. New therapeutic strategies targeting the gut microbiome include probiotics, prebiotics, synbiotics, postbiotics, antibiotics, and FMT. Clinical trials to date indicate significant potential for these therapies in the symptomatic treatment of gastrointestinal dysfunction and the reduction of motor and non-motor symptoms. Changes in the gut microbiota, which involve eliminating or inhibiting the activity of bacteria that degrade L-DOPA to dopamine, are a key therapeutic target for improving symptoms responsive to dopaminergic therapy. However, large-scale clinical trials of PD treatments based on the gut–microbiome–brain axis theory are still lacking. Further research is required to investigate the potential benefits of treatments from a microbiota–gut–brain axis perspective in both animal and human models. This research could help us better understand the mechanisms of intestinal inflammation and microbially induced systemic inflammation, particularly at the molecular level.

## 8. Limitations and Future Development Directions

This review has several limitations, focusing on the Braak hypothesis and one subtype of the disease, suggesting that pathological changes may arise in the gut many years before the appearance of brain pathology. Several studies have challenged this hypothesis [264,265,266]. Criticisms include the fact that not all PD patients present with clinical stages and patterns of Lewy body pathology consistent with the Braak model [45,266]. Neuropathological evidence from autopsy does not unequivocally support the gut hypothesis, necessitating further research and suggesting that other models of Lewy body pathology propagation are also possible [264]. Alternative hypotheses suggest heterogeneity of pathogenesis, distinguishing two subtypes of PD: “brain first” and “body first” [11]. Imaging studies also show only partial concordance with Braak stages, suggesting selective neuronal vulnerability rather than strictly sequential disease propagation [265]. Due to the focus on the gut-to-brain axis (the direction of PD spread), analysis of other models is beyond the scope of this review, but it also represents a valuable avenue for further research. This review is also limited by translational gaps between animal model studies and clinical trials in PD patients. Therefore, the analysis of mechanisms related to GBA at some levels was presented only based on general neurobiology. Furthermore, the mechanistic pathways involving neurotransmitters in the gut–brain axis are still not fully understood, so drawing definitive conclusions about causality and therapeutic targets seems too speculative. Finally, there may be a lack of discussion on the challenges of standardizing or personalizing therapy based on the discussed interventions. Therapies targeting the gut microbiota in PD show promise, particularly in improving motor symptoms and gastrointestinal function, such as constipation, as evidenced by meta-analyses of randomized controlled trials demonstrating benefits in motor outcomes and bowel movements. In the case of FMT specifically, a particularly promising approach has emerged, leveraging the potential of this potential therapy to restore microbial balance and alleviate motor and non-motor symptoms via the gut–brain axis. However, clinical evidence is still preliminary and requires further rigorous testing. Future research directions should focus on large-scale, well-designed clinical trials to confirm efficacy, clarify optimal treatment regimens (dosage and duration), understand long-term effects, and explore personalized approaches based on individual microbiome profiles. Integrating multiomics technologies and mechanistic studies also appears crucial to unraveling the complex gut–brain interactions and developing novel disease-modifying strategies targeting the microbiome in Parkinson’s disease.

## Figures and Tables

**Table 1:** The influence of changes in gut microbiota composition on the Gut-Brain Axis in PD.

Microbial Species	Neurotransmitter Affected	Mechanism	Level of Evidence	References
*Enterococcus faecalis*	Dopamine	Tyrosine decarboxylase converts L-DOPA → dopamine, reducing drug bioavailability	Level IV (preclinical mechanistic + ex vivo human microbiota studies)	[71]
*Eggerthella lenta*	Dopamine (indirect)	Dehydroxylation of dopamine metabolites and L-DOPA	Level IV (preclinical mechanistic + ex vivo human microbiota studies)	[72,73]
*Clostridium* spp.	Serotonin	SCFA-mediated stimulation of enterochromaffin cells → serotonin synthesis	Level IV (preclinical mechanistic + germ-free mouse models)	[74]
*Lactobacillus rhamnosus*	GABA	GABA production via glutamate decarboxylase; vagus-mediated signaling to CNS	Level V → IV (preclinical mechanistic + animal behavioral model)	[75]
*Bifidobacterium dentium*	GABA	Direct GABA biosynthesis and secretion	Level V → IV (mechanistic + animal model evidence)	[76]
Multiple gut taxa (*Bacteroides* spp., *Parabacteroides* spp., *Escherichia coli* (selected strains)	GABA	Expression of GABA metabolic pathways in human microbiome	Level IV-V (human microbiome analysis + in vitro validation)	[77]
*Turicibacter sanguinis*	Serotonin (host interaction)	Serotonin transporter-like system modulates host–microbe signaling	Level IV (animal + host interaction)	[78]
Multiple gut taxa (*Bacteroidetes*, *Firmicutes*, *Lactobacillus*, *Bifidobacterium*, *Akkermansia* spp.)	GABA, serotonin, dopamine, norepinephrine, glutamate	Modulation of neurotransmitter synthesis, metabolism, and receptor expression; effects mediated via vagus nerve, immune signaling, SCFA production, tryptophan metabolism	Level IV (human observational studies)	[79]

## Data Availability

Data sharing is not applicable to this article as no datasets were generated or analyzed during the current study.
